# A nationwide assessment of the burden of healthcare-associated infections and antimicrobial use among surgical patients: results from Serbian point prevalence survey, 2017

**DOI:** 10.1186/s13756-021-00889-9

**Published:** 2021-03-06

**Authors:** Vesna Šuljagić, Milica Bajčetić, Vesna Mioljević, Gorana Dragovac, Biljana Mijović, Ivana Janićijević, Zorana Đorđević, Gordana Krtinić, Violeta Rakić, Ivana Ćirković, Vladimir Nikolić, Ljiljana Marković-Denić

**Affiliations:** 1 Faculty of Medicine of Military Medical Academy, University of Defence, Belgrade, Serbia; 2 Faculty of Medicine, University of Belgrade, Belgrade, Serbia; 3 Clinical Centre of Serbia, Belgrade, Serbia; 4 Institute of Public Health of Vojvodina, Novi Sad, Serbia; 5 Faculty of Medicine, University of Novi Sad, Novi Sad, Serbia; 6 Faculty of Medicine, University of East Sarajevo, Foča, Bosnia and Herzegovina; 7 General Hospital of Čačak, Čačak, Serbia; 8 Institute of Public Health of Niš, Niš, Serbia; 9 Clinical Center of Kragujevac, Kragujevac, Serbia; 10 General Hospital of Subotica, Subotica, Serbia; 11 Institute of Public Health of Serbia, Belgrade, Serbia

**Keywords:** Healthcare-associated infections, Antibiotics, Point prevalence survey

## Abstract

**Background:**

As the only non-European Union (EU) country, Serbia participated in a second point prevalence survey of healthcare-associated infections (HAIs) and antimicrobial use (AMU) organized by the European Centre for Disease Prevention and Control (ECDC) in the EU countries. Here, we aimed to estimate the prevalence of HAI and AMU in patients who had recently undergone a surgery and to compare risk profile, HAI rates, and AMU among surgical patients and non-surgical patients.

**Methods:**

A national PPS was performed in 65 Serbian acute-care hospitals, in November 2017. In this paper, the data of 61 hospitals for adult acute-care were analyzed. To ensure the comparability of study design we used the Serbian translation of ECDC case definitions and ECDC PPS protocol. The trained infection control staff, led by a hospital coordinator, reviewed medical records to identify HAI active at the time of the survey and AMU. Only inpatients admitted to the ward before 8 a.m. on the day if the survey were included.

**Results:**

A total of 12,478 patients from 61 hospitals for adult acute-care were eligible for inclusion in this study. Significantly higher proportions of surgical patients were female, belonged to the 60-to-79 age group, and were less severely ill. Also, extrinsic factors (invasive devices, hospitalization at the ICU, and prior antibiotics therapy) were more frequent in surgical patients. Prevalence of HAIs was higher among surgical patients (261/3626; 7.2%) than among non-surgical patients (258/8852; 2.9%) (*p* < 0.0001). The highest prevalence of all HAIs was noted in patients who had kidney transplantation (4/11; 36.4%), while SSIs were the most prevalent among patients who had peripheral vascular bypass surgery (3/15; 20.0%). Non-surgical patients received treatment for community-acquired infections in significantly higher proportion (2664/8852; 64.3) (*p* < 0.001). Surgical prophylaxis for more than 1 day was applied in 71.4% of surgical patients.

**Conclusion:**

We have provided an insight into the burden of HAIs and AMU among Serbia acute-care hospitals, and highlighted several priority areas and targets for quality improvement.

## Background

Healthcare-associated infections (HAI) are serious complications impacting morbidity, mortality, and healthcare costs worldwide. The inappropriate use of antibiotics, and the consequent increase in antimicrobial resistance of HAI pathogens, can compromise the outcome of treatment [1]. The point prevalence surveys (PPS) that use the standardized methodology and consensus definitions of infection are relatively inexpensive and easy tools of data collection and can provide valuable information [2]. Different high-income countries [3–7] and low-and middle-income countries (LMICs) [8–10] outside Europe conducted PPSs in recent years.

In 2016–2017, the European Centre for Disease Prevention and Control (ECDC) organized the second European PPS on HAI and antimicrobial use in acute care hospitals. Twenty-eight countries participated in this survey [11, 12].

Three national PPSs were conducted in Serbia, in 1998, 2005, and 2010 years, the latest one using the ECDC methodology for the first EU PPS. Although Serbia is not an EU country, we participated in the second European PPS, using the same methodology and the same HAI definitions.

Patients undergoing surgical procedures are at increased risk of acquiring HAIs [13]. Examining the current distribution and frequency of HAIs and antimicrobial use (AMU) surgery is of paramount importance for guided risk-reduction interventions [14]. A comprehensive understanding of the epidemiology and burden of HAI in surgical patients is critical for taking preventive measures [15]. Therefore, based on data from the last PPS in our country, we aimed at estimating the prevalence of HAI and AMU in patients who recently had a surgery in Serbian acute-care hospitals. Furthermore, risk profile, HAI rates, and AMU in this population were compared to the ones of non-surgical patients.

## Methods

### Study design and data collection

Point prevalence survey (a cross-sectional study), within the European PPS, was conducted in all 65 acute-care hospitals in Serbia, including two private hospitals. Hospitals participated on voluntary basis.

In brief**,** to ensure comparability of study design, we used the Serbian translation of ECDC case definitions and ECDC PPS protocol (version 5.3) [16]**.** Before the study took place in November 2017, several training courses were organized for infection control staff and at least one coordinator in each hospital to outline the case definitions and survey protocol. This trained infection control staff, led by the hospital coordinator, reviewed medical records to identify HAI active at the time of the survey.

Data were collected in a single day, in one ward, with a maximum time frame of 2 weeks in one hospital, and within one month for the whole national survey. The first hospital started its survey on October 26, and the last day of the survey at the hospital which was the last to start, was November 26, 2017.

All public acute-care hospitals were invited to participate in the survey by an official letter sent to them by the Ministry of Health. Besides, both of the two private hospitals in Serbia applied for the study, so the total number of hospitals that participated in the fourth PPS within the second EU PPS was 65.

**Fig. 1:**
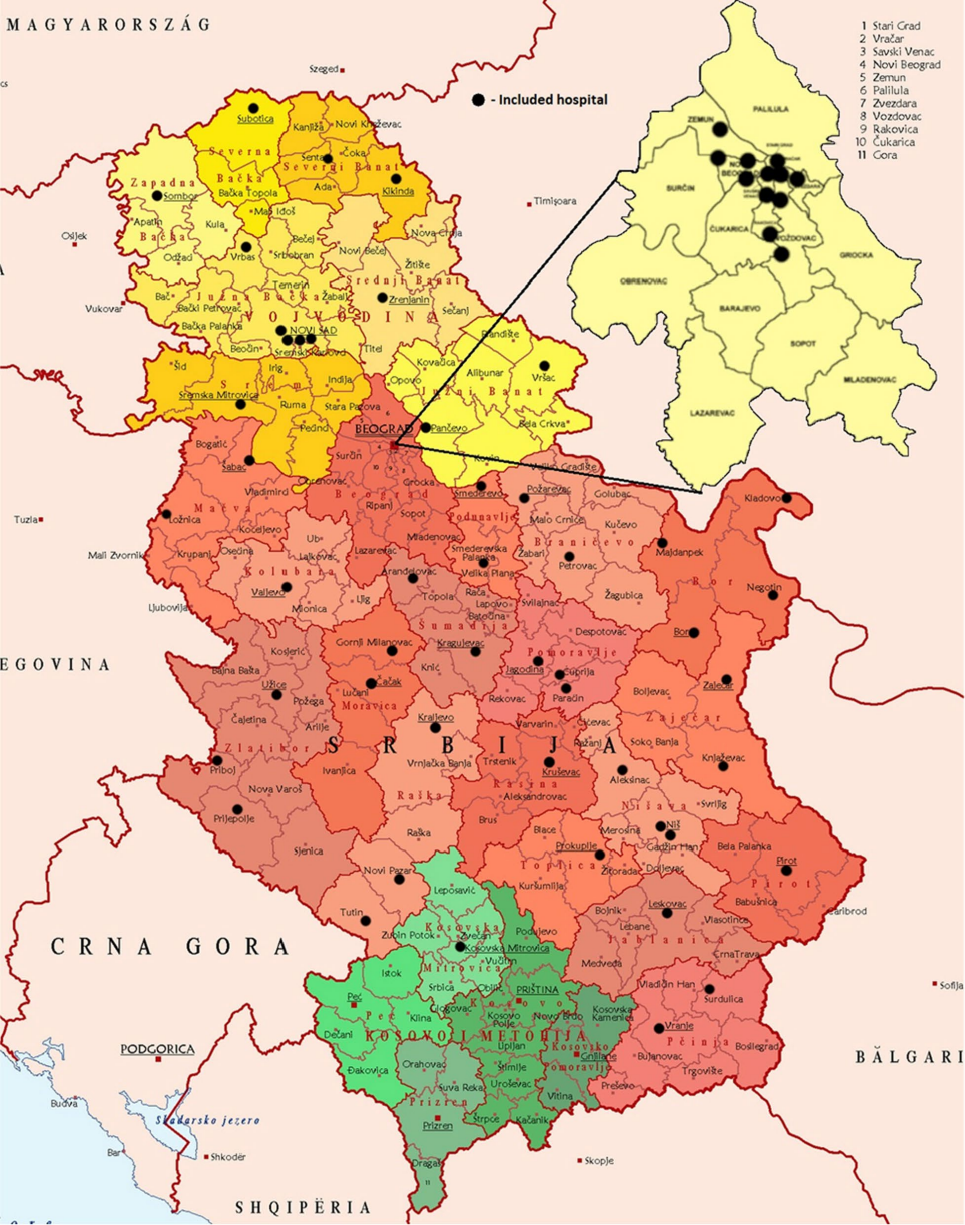
The geographical distribution of hospitals included in Serbian point prevalence survey

In this paper, we have sought to determine the antibiotic consumption and risk factors (RF) for HAIs in adult patients. Consequently, for the current analyses, data from four hospitals (two pediatric ones, a long-term care one, and a psychiatric hospital) were excluded. Therefore, the data of 61 hospitals for adult acute-care were here analysed. Figure 1 shows the geographical distribution of the included hospitals.

Only inpatients admitted to the ward before 8 a.m. on the day of the survey, and not discharged from the ward during the conducting of the survey, were included. Patients in the emergency room, dialysis patients, patients in outpatient departments, and day patients (day cases) who did not stay overnight in the hospital were excluded.

Data collection included variables at the national, hospital, ward, and patient level. Data of hospitals included information about the number of hospital beds and the number of intensive-care units (ICU) beds, a specificity of hospital wards, type of hospital (primary, secondary, tertiary healthcare level), and hospital ownership. According to the number of beds, hospitals were presented in four categories: small (< 360 beds), medium-sized (360–575 beds), large (575–1100 beds), and very large hospital (> 1100 beds).

The standard, patient-based, protocol was used and denominator data were collected for each patient. Patient data were collected using the ECDC questionnaire which in the first part included age, sex, date of hospital admission, survey date, the specialty of the ward where the patient was hospitalized, surgery (type and date), and severity of underlying medical conditions presented by McCabe score.

Surgical patients were defined as patients who had undergone a surgery within the past 30 days, or within the past 90 days in the case of implant surgery. Non-surgical patients were designated as patients not having surgery. Surgery was defined as a procedure performed primarily for therapeutic reasons where an incision is made (not just a needle puncture), with breach of mucosa and/or skin—not necessarily in the operating room. Three categories of surgery were defined: National Healthcare Safety Network (NHSN) surgery, minimally invasive/non-NHSN surgery, and unknown type of surgery [16]. According to the ECDC protocol, the unknown type of surgery referred to the unregistered type of surgical operation in the patient's protocol. According to the McCabe score [16] patients were classified as patients with the non-fatal disease (expected survival at least 5 years), or with an ultimately fatal disease (expected survival between 1 and 5 years), or with a rapidly fatal disease (expected death within 1 year).

The second part of the questionnaire referred to information on invasive devices: central and peripheral vascular catheter, urinary catheter, and intubation.

If the patient was receiving antimicrobials at the time of the survey, the information about Antimicrobial Therapeutic Chemical (ACT) classes, route of administration, date of the start of antimicrobial use, indication for antimicrobial use, whether the antibiotic was altered, and the reason for the change, and dosage per day was collected. The invasive devices (urinary catheter, central venous catheter, mechanical ventilation), intensive care units (ICU) hospitalization, and prior antibiotics therapy were considered as extrinsic risk factors.

The last part of the questionnaire referred to the presence of active healthcare-associated infections (HAI). According to the ECDC definitions, all HAIS were classified in one out of 14 groups [16].

The Ministry of Health sent an invitation to all acute-care hospitals to participate in this national survey. Hospitals participated on voluntary basis. Each participating hospital signed approval to participate in the study. The additional approval was not deemed necessary, because patient data were collected anonymously, according to the ECDC PPS protocol. Each hospital had its code which was known only to researchers at the hospital and to the small study team at the national level. The results of the entire study were presented at the national level in the publication issued by the Ministry of Health, while the results of the hospitals were presented under a code.

### Statistical analysis

The local infection-control team entered the data into ECDC’s HelicsWin.Net software that allows anonymous data entry and validation. Additional data analysis was performed using SPSS, version 17 (SPSS, Inc, Chicago, IL). Results were expressed as the mean ± SD or as the proportion of the total number of patients. The prevalence of patients with at least one HAI was calculated as a percentage of patients with at least one HAI divided by a total number of patients. The prevalence of all HAI was calculated as a percent of all HAI divided by the number total number of patients.

The χ^2^ test or Fischer exact test was used for categorical variables and relative risk, and their corresponding 95% confidence intervals (CI) were calculated. For parametric continuous variables, mean values were compared using the Student *t* test. For nonparametric continuous variables, the Mann–Whitney *U* test was used. RF independently associated with HAI were identified by stepwise logistic regression analysis of variables selected by univariate logistic regression analysis (ULRA), with a limit for entering and removing categorical variables from the model at *p* = 0.05.

## Results

A total of 12,478 patients from 61 hospitals for adult acute-care were eligible for inclusion in this study. Out of all patients, 5565 (44.6%) were hospitalized in various internal-medicine wards, 4896 (39.2%) in surgical wards, 1342 (10.8%) in gynecology/obstetrics wards, 521 (4.2%) in Intensive Care Unit (ICU), 98 (0.8%) in geriatrics, and 0.4% in other mixed wards.

The median age of all patients was 60.6 years (from 18 to 99 years), and 6634 (53.1%) of them were female. Out of all patients, 3626 (29.1%) were operated on according to the study definitions, out of which 3605 (28.9%) during the current hospitalization. The main characteristics of surgical patients and non-surgical patients are presented in Table 1. Significantly higher proportions of surgical patients were female, belonged to the 60-to-79 age group, and they were less severely ill, according to the McCabe score. Also, selected extrinsic factors (invasive devices, hospitalization at the ICU, and prior antibiotics therapy) were more frequent in these patients. A higher proportion of operated patients were hospitalized in tertiary hospitals, classified as very large hospitals according to the number of beds.

**Table 1:** Characteristics of surgical and non-surgical patients, Serbian PPS study, 2017

	Surgical patients N = 8852	Non-surgical patients N = 3626	*p* value*
	N (%)	N (%)	
Intrinsic factors			
Sex			< 0.0001
Female	4610 (52.1)	2024 (55.8)	
Male	4242 (47.9)	1602 (44.2)	
Age group			< 0.0001
< 40 years	1171 (13.2)	792 (21.8)	
40–59	1921 (21.7)	844 (23.3)	
60–79	4703 (53.1)	1683 (46.4)	
> 80	1057 (11.9)	307 (8.5)	
McCabe classification			< 0.0001
Nonfatal	6831 (77.2)	2913 (80.3)	
Fatal within 5 years	517 (5.8)	178 (4.9)	
Fatal within 1 year	1205 (13.6)	485 (13.4)	
Unknown	299 (3.4)	50 (1.4)	
Extrinsic factors			
Invasive devices			
Urinary catheter	1591 (18.0)	1287 (35.5)	< 0.0001
Central venous catheter	254 (2.9)	378 (10.4)	< 0.0001
Mechanical ventilation	127 (1.4)	141 (3.9)	< 0.0001
Exposure to intensive care	283 (3.2)	238 (6.6)	< 0.0001
Prior antibiotics therapy	2850 (32.2)	2435 (67.2)	< 0.0001
Hospital factors			
Level of hospital health care			< 0.0001
Secondary	4845 (54.7)	1631 (45.0)	
Tertiary	4007 (45.3)	1995 (55.0)	
Hospital size			< 0.0001
Small (< 360 beds)	2214 (25.0)	710 (19.6)	
Medium (360–575)	2371 (26.8)	918 (25.3)	
Large (575–1100)	2161 (24.4)	849 (23.4)	
Very large (> 1100)	2106 (23.8)	1149 (31.7)	

*Chi-square or Fisher exact test, if appropriate

### Healthcare-associated infections

**Fig. 2:**
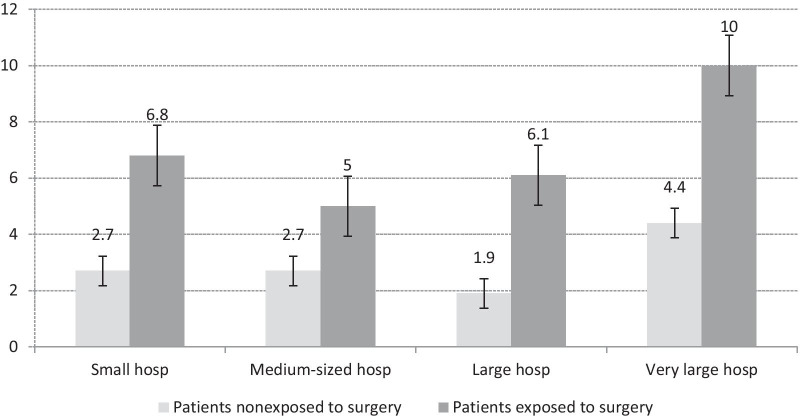
HAI prevalence among surgical and non-surgical patients according to the hospital size

A total of 519/12,478 (4.1%) adult patients included in the PPS had at least one HAI. The total number of HAIs was 548, so the prevalence of all HAIs was 4.4%. Prevalence of HAIs was higher among surgical patients (261/3626; 7.2%) than among non-surgical patients (258/8852; 2.9%) (*p* < 0.0001). Prevalence of HAI was higher in operated patients across all four hospital size categories (Fig. 2).

The most frequent ones were urinary tract infections (130/548; 23.7%), followed by SSI (125/548; 22.8%), pneumonia/lower respiratory tract infections (113/548; 20.6%), *Clostridioides difficile* (*C. difficile*) infections (62/548; 11.3%), and bloodstream infections (47/548; 8.6%).

The prevalence of SSI among patients who underwent surgery was 3.4% (125/3626). Out of all patients with SSI, 83.2% (104/125) were operated on during current hospitalization after admission, while others (16.8%) were operated on during a previous hospitalization. For 84.8% of SSI, microorganisms were reported. The most frequently identified bacteria in SSI were *Acinetobacter* spp. (14.7%), *Klebsiella* spp. (11.1%), *Staphylococcus aureus* (10.6%) and *Pseudomonas aeruginosa* (9.6%).

Prevalence of HAI other than SSI was 5.0% (181/3626) among surgical patients and 4.1% among the non-surgical ones. The risk of HAI other than SSI was significantly higher among operated patients (OR = 1.35; 95% CI = 1.09–1.66; *p* = 0.006). There was no difference between two patient groups concerning diarrhoea caused by *C. difficile*: prevalence among patients who underwent surgery was 0.4% (13/3626) and prevalence among non-operated patients was 0.6% (49/8852) (OR = 1.55; 95% CI = 0.84–2.8, *p* = 0.163).

The prevalence of patients with at least one HAI and SSIs, as well as the prevalence of patients taking at least one antibiotic are presented in Table 2. The highest prevalence of all HAIs was noted in patients who had a kidney transplantation (36.4%), while SSIs were the most prevalent among patients who had peripheral vascular bypass surgery (20.0%).

**Table 2:** Prevalence of healthcare-associated infections, surgical site infections, and antibiotics according to the operative procedures, Serbian PPS study, 2017

Operative procedure according NHSN	Number of patients who had an operative procedure	Prevalence		
		Patients with at least one HAI	Patients with SSI	Patients taking at least one antibiotic
		N (%)		
Kidney transplant	11	4 (36.4)	1 (9.1)	10 (90.9)
Peripheral vascular bypass surgery	15	4 (26.7)	3 (20.0)	12 (80.0)
Coronary artery bypass graft with chest incision only	12	3 (25.0)	0 (0.0)	8 (66.7)
Craniotomy	100	23 (22.0)	0 (0.0)	83 (83.0)
Gastric surgery	66	14 (21.2)	6 (9.1)	56 (84.8)
Shunt for dialysis	6	1 (16.7)	0 (0.0)	1 (16.7)
Carotid endarterectomy	12	2 (16.7)	1 (8.3)	11 (91.7)
Kidney surgery	80	12 (15.0)	7 (8.8)	72 (90.0)
NHSN non-defined	171	25 (14.6)	25 (14.6)	106 (62.0)
Colon surgery	153	22 (14.4)	11 (7.2)	111 (72.5)
Abdominal aortic aneurysm repair	28	4 (14.3)	1 (3.6)	26 (92.9)
Limb amputation	106	15 (14.2)	11 (10.4)	71 (67.0)
Small bowel surgery	46	6 (13.7)	4 (8.7)	35 (76.1)
Neck surgery	16	2 (12.5)	1 (6.3)	12 (75.0)
Coronary artery bypass graft with both chest and donor site incisions	67	8 (11.9)	4 (6.0)	47 (70.1)
Spinal fusion	27	3 (11.1)	1 (3.7)	16 (59.3)
Spleen surgery	9	1 (11.1)	0 (0.0)	8 (88.9)
Thoracic surgery	56	6 (10.7)	4 (7.1)	34 (60.7)
Cardiac surgery	57	6 (10.5)	0 (0.0)	32 (56.1)
Prostate surgery	76	7 (9.2)	5 (6.6)	70 (92.1)
Vaginal hysterectomy	33	3 (9.1)	1 (3.0)	28 (84.8)
Bile duct, liver or pancreatic surgery	65	5 (7.7)	2 (3.1)	55 (84.6)
Laminectomy	26	2 (7.7)	1 (3.8)	20 (76.9)
Open reduction of fracture	220	16 (7.3)	9 (4.1)	157 (71.4)
Exploratory laparotomy	68	4 (5.9)	2 (2.9)	53 (77.9)
Abdominal hysterectomy	58	3 (5.2)	3 (5.2)	45 (77.6)
Hip prosthesis	269	10 (3.7)	6 (2.2)	176 (65.4)
Non-NHSN/minimal surgery	1017	34 (3.3)	10 (1.0)	574 (56.4)
Knee prosthesis	62	2 (3.2)	2 (3.2)	44 (71.0)
Gallbladder surgery	106	3 (2.9)	2 (1.9)	69 (65.1)
Rectal surgery	35	1 (2.9)	1 (2.9)	25 (71.4)
Cesarean section	241	6 (2.5)	3 (1.2)	169 (70.1)
Ovarian surgery	41	1 (2.4)	0 (0.0)	29 (70.7)
Herniorrhaphy	123	2 (1.6)	1 (0.8)	87 (70.7)
Appendix surgery	29	0 (0.0)	0 (0.0)	24 (82.8)
Breast surgery	72	0 (0.0)	0 (0.0)	32 (44.4)
Liver transplant	2	0 (0.0)	0 (0.0)	2 (100.0)
Pacemaker surgery	11	0 (0.0)	0 (0.0)	5 (45.5)
Refusion of spine	4	0 (0.0)	0 (0.0)	4 (100.0)
Thyroid and/or parathyroid surgery	27	0 (0.0)	0 (0.0)	13 (48.1)
Ventricular shunt	3	0 (0.0)	0 (0.0)	3 (100.0)
Total	3626	519 (4.2)	125 (1.0)	2435 (67.2)

*NHSN* National Healthcare Safety Network, *HAI* healthcare-associated infections, *SSI* surgical site infections

The multivariate logistic regression analysis (MLRA) revealed that male sex, ultimately and rapidly fatal underlying disease according McCabe score, tertiary hospital level and presence of urinary catheter (UC), central venous catheter (CVC), and mechanical ventilation (MV) were independent RFs for HAI, while antibiotic therapy was a protective factor for HAI (Table 3).

**Table 3:** Factors associated with healthcare-associated infections according to the results of univariate and multivariate logistic regression analysis

Variables	Without HAI	With HAI	Logistic regression model			
			Univariate		Multivariate	
	n (%)	n (%)	OR (95%CI)	*p*	OR (95%CI)	*p*
Male sex	5546 (46.4)	298 (57.4)	1.56 (1.31–1.86)	< 0.001	1.30 (1.08–1.58)	0.006
Age > 60 years	7391 (61.8)	359 (69.2)	1.18 (1.07–1.29)	0.001	–	–
McCabe (nonfatal vs others)	9445 (79.0)	299 (57.6)	2.76 (2.31–3.31)	< 0.001	1.44 (1.17–1.77)	< 0.001
Hospital level						
Secondary	6268 (52.4)	208 (40.1)	ref			
Tertiary	5691 (47.6)	311 (59.9)	1.65 (1.38–1.97)	< 0.001	1.30 (1.06)	0.10
Hospital size						
Small hosp.	2816 (23.5)	108 (20.8)	Ref			–
Large hosp.	2916 (24.4)	94 (18.1)	1.40 (1.17–1.67)	< 0.001	–	
Surgery	3365 (28.1)	261 (50.3)	2.58 (2.16–3.08)	< 0.001	–	–
ICU	438 (3.7)	83 (16.0)	5.01 (3.89–6.45)	< 0.001	–	–
Urinary catheter	2574 (21.5)	304 (58.6)	5.15 (4.31–6.17)	< 0.001	1.78 (1.45–2.19)	< 0.001
Mechanical ventilation	182 (1.5)	86 (16.6)	12.85 (9.77–16.90)	< 0.001	2.38 (1.70–3.30)	< 0.001
CVC	486 (4.1)	146 (28.1)	9.24 (7.48–11.42)	< 0.001	2.33 (1.79–3.06)	< 0.001
Antibiotic therapy	4778 (40.0)	507 (97.7)	0.016 (0.009–0.28)	< 0.001	0.23 (0.13–0.41)	< 0.001

*McCabe* McCabe classification, *ICU* intensive care units, *CVC* central venous catheter

### Antibiotics use

A total of 5285/12,478 (42.4%) adult patients included in the PPS, received at least one antibiotic. Significantly higher proportion of surgical patients received antibiotics (2435/3626; 67.2%) in comparison to the non-surgical patients (2850/8852; 32.2%) (*p* < 0.0001). A total of 3477 antibiotics were reported for operated patients which were 1.43 antimicrobial per patient, while 4145 antibiotics were administered to 2850 non-operated patients (1.45 antimicrobial per patient).

Antibiotics were most frequently prescribed for treatment of infections: 40.5% for community-acquired infections, 9.8% for healthcare-associated infections, and 1.2% for infection acquired during the previous stay in long-term care facilities. Non-surgical patients received treatment in a significantly higher proportion for community-acquired infections (64.3% vs. 12.1%). Surgical prophylaxis for more than 1 day was applied in 71.4% of patients who underwent surgery (Table 4).

**Table 4:** Indication for antimicrobial use, Serbian PPS study, 2017

Antimicrobial indication	Non-surgical patients	Surgical patients	Total
	N (%)	N (%)	N (%)
Treatment of community-acquired infection	2664 (64.3)	420 (12.1)	3084 (40.5)
Treatment of healthcare-acquired infection	380 (9.2)	370 (10.6)	750 (9.8)
Treatment of infection acquired in long term care facilities	61 (1.5)	33 (0.9)	94 (1.2)
Surgical prophylaxis		2089 (60.1)	2191 (28.7)
1 dose		281 (13.5)	298 (13.6)
1 day	N/A	317 (15.2)	337 (15.4)
> 1 day		1491 (71.4)	1556 (71.0)
Medical prophylaxis	721 (17.4)	386 (11.1)	1107 (14.5)
Other	37 (0.9)	51 (1.5)	88 (1.2)
Unknown	180 (4.4)	128 (3.7)	308 (4.0)

**Fig. 3:**
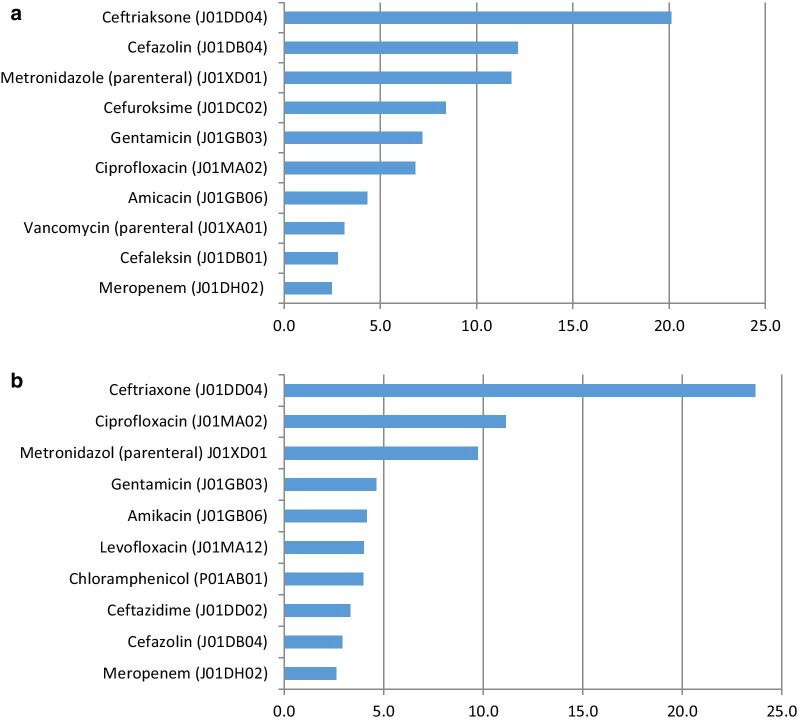
The most often prescribed antibiotics to **a** surgical patients, Serbian PPS study, **b** non-surgical patients, Serbian PPS study

The antimicrobial agents most often prescribed to surgical and non-surgical patients are presented in Fig. 3.

Ceftriaxone was the most frequently prescribed antibiotic in both groups of patients. Even more important, it was the most prescribed antibiotic in surgical prophylaxis (21.3% of all antibiotics prescribed for this indication). Cefazolin and cefuroxime made 18.4 and 12.9% respectively of all antibiotics prescribed in surgical prophylaxis (not presented in the table).

## Discussion

Numerous studies on the prevalence of HAI and AMU have been conducted worldwide over the past 10 years [3–11, 17–21]. However, there are still very few studies that define the study populations by recently having a surgical procedure, and not according to the ward they are hospitalized at on the study day [22]. This is the fourth nationwide prevalence survey of HAI and antibiotic consumption, conducted within the second European PPS, aimed to compare surgical patients with non-surgical patients in Serbian acute-care hospitals. We found that 29% of all adult patients had undergone a recent surgical procedure and the prevalence of HAI in that group of patients was almost two and half times higher than in non-operated patients. The most frequent infections were UTIs and SSIs. At least one antibiotic was taken by 42% of surveyed patients, mainly for the treatment of community-acquired infections (40%) and for surgical prophylaxis (29%).

Firstly, this study finds that according to the ULRA surgery was a significant RF for HAI (*p* < 0.001, OR: 2.38, 95% CI: 2.16–3.08), but it was not a significant independent RF according to MLRA (OR = 1.01 95% CI 0.829–1.238). The first Singapore PPS showed that surgery since admission detected in 23.7% patients in acute care hospitals and was independent RF for HAI [6], while multicenter PPS, conducted in Switzerland during 2016 showed that NHSN surgery was not independent RF for HAI (ULRA-OR: 1.77, 95% CI 0.89–3.54, *p* 0.106 and MLRA-OR 1.41, 95% CI 0.75–2.67, *p* 0.288) [19].

Secondly, in Serbian acute adult patients, the prevalence of HAI was 7.2% and 2.9% in surgical and non-surgical patients respectively. National PPS of HAI and antimicrobial prescribing done in Scotland noted similar results (4.0% vs. 6.5%) [20]. This characteristic observed across all four hospital size categories (Fig. 1) is not a new phenomenon and has been reported before [22]. The reasons for the difference are likely to be multifactorial. Sax et al. showed, importantly, that patients admitted to larger hospitals had a greater number of comorbidities. Also, prolonged hospital and ICU stay, medical devices and drugs use were more frequent in larger hospitals [23]. One of the reasons for the higher HAI prevalence in surgical patients would be the occurrence of SSI, the second most frequent HAI in Serbian PPS, with a prevalence of 3.4%. Furthermore, the most frequent HAI in Serbian patients were urinary tract infections (UTI) (130/548; 23.7%), SSIs (125/548; 22.8%) and pneumonia/lower respiratory tract infections (113/548; 20.6%). On the contrary, infections not associated with devices or operative procedures—including *C. difficile* infections and other gastrointestinal infections, and non-ventilator-associated pneumonia—accounted for approximately half of all HAI in prevalence surveys conducted in US hospitals in 2015 [3]. This distribution of HAI, different from the one established by a survey conducted in 2011, was explained by Magill et al. with experience which had shown that HAI, specifically UTI and SSI, could be prevented by national public-health focus and evidence-based interventions [3, 24, 25]. The significant differences in the prevalence of UC, CVCs, and the use of MV between both groups of our patients are shown in Table 1. MLRA revealed that the presence of invasive medical devices (UC, CVC, and MV) was independent RF for HAI in Serbian acute adult patients. To stop the spread of HAIs, more attention needs to be paid to the role of invasive medical devices. Implementing a quality-management system seemed like the most effective way to prevent a significant number of these infections. WHO encouraged countries with a weak quality-management system to hekp healthcare professionals use invasive medical devices in a manner that is fair, consistent, and effective [26].

Thirdly, the highest prevalence of all HAIs was noted in patients who had kidney transplantation (36.4%). This is an expected result because immunosuppression is one of the most important RF for infections, including HAI in this group of patients. Monlezun et al. [27] reported that 48.7% of renal transplant patients experienced at least one post-transplant infection. Similar findings were reported by U.S. Renal Data System which showed that UTIs were the most common bacterial infections requiring hospitalization in kidney transplant recipients, followed by pneumonia, SSI, and bloodstream infections [28]. Moreover, SSIs were the most prevalent among patients who had peripheral vascular bypass surgery (20.0%). This is not a surprising result since, in a few prospective analytic and experimental studies, SSI was identified as one of the most common postoperative complications after vascular reconstruction, particularly lower extremity bypass procedures [29, 30]. The published incidence of SSI after lower extremity bypass procedure varied from 4.8 [31] to 22.8% [32].

In Ghana's PPS for only 10% of SSI, the microorganism was reported with a dominance of gram-negative bacteria, while in Singapore’s PPS the most frequently identified bacteria in SSI were *S. aureus* (17.5%), followed by *P. aeruginosa* (14.6%), *Escherichia coli* (9.5%) and *Acinetobacter* spp. (4.4%) [6]. We registered microorganisms in 84.8% of SSI. Also, we observed different distribution of bacteria causing SSI—*Acinetobacter* spp. (14.7%), *Klebsiella* spp. (11.1%), *S. aureus* (10.6%) and *P. aeruginosa* (9.6%).

The third Slovenian national HAI PPS detected *C. difficile* gastrointestinal infections in almost half of the identified gastrointestinal infections [21]. Altogether, our data show that gastrointestinal HAIs were in the fourth place in frequency (13.8% of all HAI) and that there was no difference in the prevalence of *C. difficile* gastrointestinal infections in patients who recently had a surgery compared with non-operated patients (OR = 1.55 95% CI = 0.84–2.8, *p* = 0.163). Some studies provided evidence that although surgical patients tend to suffer more severe CDIs than medical patients, overall they still do better than medical ones [33, 34].

The present study found that exposure to at least one antimicrobial agent was registered in 42.4% of hospitalized patients. That was lower than the antimicrobial use prevalence reported in China's prevalence survey (49.63%) [7] and higher than reported in Japan's prevalence survey (33.5%) [5]. Compared to other PPS in European countries, Serbian patients received at least one antimicrobial agent (42.4%), which is below average values for hospitalized patients in Bulgaria (45.2%), Cyprus (45.8%), Greece (55.6%), Italy (44.5%), and Spain (46.3%), but above the EU average (30.5%) [12]. Similar to the study performed by Sax et al. [22], in our study surgical patients received antimicrobial agents significantly more frequently than non-surgical patients. Moreover, non-surgical patients received antimicrobial agents significantly more frequently for community-acquired infections (64.3% vs. 12.1%). We can assume that a higher rate of antibiotics prescriptions for community-acquired infections is a consequence of the fact that significantly higher proportions of non-surgical patients (65%) were patients over the age of 60 with comorbidities and a clinical history of the infection treatment before the hospitalization. The second PPS of HAI and antimicrobial use in European acute-care hospitals showed that the most common indication for prescribing antimicrobials was the treatment of community-acquired infection in 69.8% [12].

Antibiotics prophylaxis is defined as the administration of a single dose of the effective antimicrobial agent prior to the exposure with possible contamination, i.e., surgery, to decrease the risk of postoperative infections [35]. A single dose of antibiotic is recommended to be given 120 to 60 min before surgical incision. Moreover, the recently published guidelines of the Centers for Disease Control and Prevention (CDC) restrict any additional prophylactic antibiotics after the completion of the clean and clean-contaminated surgical procedures [36]. In contrast to data from UK Scotland, Finland, Luxemburg, Germany, Austria [12], more than 70% of our surgical patients received surgical prophylaxis for longer than 1 day.

Regarding the antibiotics administration dosing regimen and duration of antibiotics prophylaxis, the appropriate antimicrobial selection also plays a role in minimizing possible risks of bacterial resistance and *C. difficile* gastrointestinal infections. It has a secondary favorable effect of potentially minimizing infection rates. Cefazolin, a first-generation cephalosporin, provides adequate coverage against most of the organisms causing postoperative infections in patients with no history of beta-lactam allergy, and a history of MRSA infection. It causes minimal allergic reactions and side effects, achieves adequate tissue levels, and is relatively inexpensive. The presence of these benefits makes cefazolin the most appropriate perioperative antibiotic prophylaxis agent for the majority of surgical procedures [35]. Overall, cefazolin was prescribed only in 18.4% of surgical patients as perioperative surgical prophylaxis. The most frequently prescribed antibiotics in both groups of our patients was ceftriaxone, a third-generation cephalosporin belonging to the “watch group” by WHO. Plachouras et al. [12] reported that ceftriaxone was in third place of the list of antimicrobial agents accounting for 75% of antimicrobial use in acute-care hospitals in the European Union/European Economic Area countries during 2016–2107, with the highest consumption registered in Bulgaria, Romania, and Serbia.

This survey has its limitations. Firstly, data collection was done by local infection-prevention and control professionals and not by the study team. However, all participating data collectors were trained by the PPS coordination committee of the Ministry of Health of Serbia before data collection and most of them had experience with performing local PPSs in the past. Secondly, we did not derive the local HAI incidence from our prevalence results as the routine applicability of the Rhame and Sudderth formulae [37]. The strength of our study is that the data are representative of Serbia as the data from 61 hospitals for adult acute-care were included. Additionally, the data on the use of antibiotics and HAI from our study contributed to the quality of the National guidelines for the rational use of antibiotics published in 2018 by the Ministry of Health of the Republic of Serbia. We hope that the implementation of the National guidelines will significantly improve antibiotics prescription politics and reduce antimicrobial resistance in the future.

## Conclusion

In summary, we provided an insight into the burden of HAIs and AMU in Serbian acute-care hospitals and highlighted several priority areas and targets for quality improvement. AMU was more frequent than the EU average, the common indication was the treatment of community-acquired infections. The MLRA revealed that male sex, ultimately and rapidly fatal underlying disease according to McCabe score, tertiary hospital level and presence of a urinary catheter, CVC, and mechanical ventilation were independent risk factors for HAI. This represents a key target intervention area for reducing HAI prevalence in Serbia.

## Data Availability

The data sets used and/or analysed in the present study are available from the corresponding author on reasonable request.

## Abbreviations

EU: European Union
PPS: Point prevalence survey
HAI: Healthcare-associated infection
AMU: Antimicrobial use
ECDC: European Centre for Disease Prevention and Control
RF: Risk factor
CI: Confidence intervals
SSI: Surgical site infection
MLRA: Multivariate logistic regression analysis
UC: Urinary catheter
CVC: Central venous catheter
MV: Mechanical ventilation
UTI: Urinary tract infection
SSI: Surgical site infection
CDC: Centre for Disease Control and Prevention
